# Congenital glucose‐galactose malabsorption: A case report with a novel *SLC5A1* mutation

**DOI:** 10.1002/ccr3.1913

**Published:** 2018-11-11

**Authors:** Manar Al‐lawama, Jumana Albaramki, Mutaz Altamimi, Hatem El‐Shanti

**Affiliations:** ^1^ Department of Pediatrics, School of Medicine The university of Jordan Amman Jordan; ^2^ Department of Pediatrics, College of Medicine University of Iowa Iowa City Iowa

**Keywords:** congenital diarrhea, glucose‐galactose malabsorption, neonate, SLCSA1 gene

## Abstract

A three‐day‐old newborn girl presented with decreased feeding and dehydration. She was sick and in shock. She had renal impairment and hypernatremia. With the resumption of breast feeding, she developed watery stools and hypernatremia. Glucose‐Galactose Malabsorption was suspected and confirmed by the presence of a likely pathogenic homozygous variant in *SLC5A1*.

## 1 INTRODUCTION

Glucose‐ galactose malabsorption (GGM; MIM606824) is a rare autosomal recessive disorder secondary to biallelic mutations in *SLC5A1* that encodes sodium/glucose co‐transporter‐1, a member of the sodium‐dependent glucose transporter (SGLT) family allowing the transfer of glucose and galactose coupled to the intracellular sodium. This co‐transporter is present in both intestinal cells and proximal renal tubules.^1^ Few hundred cases were described in the literature.^2^

Early presentation with diarrhea and dehydration in the neonatal period is characteristic.^2^ Once the disease is suspected, the diagnosis can be further supported by cessation of symptoms with fasting state and the presence of acidic stools. Hypernatremia and renal impairment are more commonly seen with GGM than with other forms of congenital diarrhea.^3^ Genetic studies are needed to support the clinical diagnosis.

## 2 CASE REPORT

A three‐day‐old full‐term newborn girl presented to the emergency room with fever, decreased feeding, and absence of urine output of one day duration. She was exclusively breastfed and was passing stools of uncertain consistency in the first 2 days. On physical examination, she was dehydrated, sick looking, febrile (38.8^○^C, axillary), tachypnic (respiratory rate: 70 breaths/min), and with signs of respiratory distress. Her oxygen saturation was 76% at room air with prolonged capillary refill time (>4 seconds). Pregnancy, labor, and delivery were uncomplicated. She was born to first cousins parents and has an older healthy sibling, and there is no significant relevant family history.

She was intubated, given normal saline bolus (20 cc/kg) and was started on dopamine and dobutamine infusions. She was admitted to the NICU of Jordan University Hospital and started on ampicillin and gentamicin after obtaining bacterial cultures. The laboratory tests showed metabolic acidosis (pH:6.8 and HCO3 of 9.4 meq/L), hypernatremia (189 meq/L), hyperkalemia (11.1 meq/L), renal impairment (Creatinine of 2.9 mg/dL and BUN of 292), and prolonged INR (2.25). She had a normal chest X‐ray.

Despite rehydration, she was still anuric and the creatinine and potassium increased which mandated a double volume blood exchange transfusion. Echocardiography revealed normal cardiac structure and renal ultrasound showed normal‐sized kidneys without hydronephrosis and an empty bladder. Her condition improved quickly, and the metabolic acidosis resolved with fluid therapy. She was extubated and came off inotropes within 24 hours. Her electrolytes normalized, but the creatinine rose to 7.3 mg/dL on hospital day # 4. She was started on peritoneal dialysis on hospital day # 5, and feeding resumed on hospital day # 7 with expressed breast milk. Blood culture came back negative. Despite continuous dialysis and anuric state, the serum sodium started to rise again. Breast milk sodium was normal.

Based on the clinical picture and the recurrence of hypernatremia with feeding, with query history of diarrhea, Glucose‐Galactose Malabsorption (GGM) was suspected. Stool was acidic with a pH of <6. There was no reported diarrhea until hospital day 10 when the baby feeds were increased. The nursing staff and residents thought she was passing urine when in fact it was watery stools. She was started on special formula (galactomine ®‐ fructose based) and her stools and sodium normalized. The baby continued on peritoneal dialysis and developed hypertension. Her illness was further complicated by intracranial hemorrhage, hydrocephalus, and seizures. She died at the age of 4.5 months.

Genomic DNA was extracted from peripheral blood from the infant and both parents. The entire coding regions of *SLC5A1* (NM_000343.3) and corresponding exon/intron boundaries(±8 bp) were sequenced by next generation sequencing (NGS) on MiSeq (Illumina, San Diego, CA, USA). Alignment and variant calling were performed using NextGENe software (SoftGenetics, State College, PA, USA). Sanger sequencing was used to provide data for bases with insufficient coverage and for validation of variants. The classification and reporting of the variants was performed according to the international recommendation.^4^

The infant was found to be homozygous for a variant (c.1006C>T; p.R336C; rs768831308) in *SLC5A1*, and both parents were found to be heterozygous for the same variant. This variant is not reported in the disease‐related literature but is described in the dbSNP without minor allele frequency (MAF) and in the ExAC database with extremely low frequency (0.00082%) and is absent in the Greater Middle East (GME) database, as well as over 3000 ethnically matched control chromosomes from existing sequences. It changes a highly conserved amino acid and is predicted to be probably pathogenic by the protein predictive software PolyPhen and SIFT.

## 3 DISCUSSION

Our patient presented on day three of life, with shock, renal impairment, and severe hypernatremia. This presentation is typical of GGM. The watery nature of stools might be mistaken for urine and might delay the diagnosis.^5^ Working diagnosis in any newborn who presents with severe dehydration early in life should always include congenital diarrhea even if the history is not clear. The presence of hypernatremia in cases of congenital diarrhea should make physicians suspect GGM particularly.^3^, ^6^ Hypernatremia is mostly due to the complete absence of water absorption.

Finding renal abnormalities is also another clue to suspect GGM as the sodium/glucose co‐transporter‐1 is present in both intestinal cells and in renal tubules. Renal abnormalities were reported in association with GGM, namely hematuria,^7^ nephrocalcinosis and renal tubular acidosis,^8^, ^9^ rickets, nephrogenic diabetes insipidus,^10^ and renal stones. Neonatal irreversible renal failure was not reported before. The severity of renal injury in our case can be explained by massive renal tubular necrosis secondary to ischemic injury from severe dehydration and decreased renal perfusion.

Central nervous system associated abnormalities were reported before with GGM.^11^ The neurological sequelae in our case were a complication of hypertension.

The identified variant is likely pathogenic since it segregates with the disorder in this family, not present, or extremely rare, in available public databases, not present in ethnically matched control chromosomes, changes a highly conserved amino acid and is predicted to be damaging by protein predictive software tools. The parents were counseled for a recurrence risk of 25% and were given the option of prenatal diagnosis, as well as preimplantation genetic diagnosis (PGD). An alternative approach is to assume that the newborn is affected and is fed a fructose‐based formula until a molecular genetic diagnosis is reached.

## CONSENT

The parents were informed and agreed on publishing the case, since no identifiers are present in the case, written consent was not taken.

## CONFLICT OF INTEREST

The authors have no conflict of interest to declare.

## **AUTHOR CONTRIBUTION**

MA: is the primary physician, made clinical diagnosis, and wrote manuscript draft. JA: is the nephrologist, provided renal care, and revised and approved manuscript. MA: is the resident on call when patient admitted, and revised and approved manuscript. HE‐S: is the geneticist, arranged investigation, interpreted results, and revised and approved manuscript.
